# Genetic mutations in RNA-binding proteins and their roles in ALS

**DOI:** 10.1007/s00439-017-1830-7

**Published:** 2017-07-31

**Authors:** Katannya Kapeli, Fernando J. Martinez, Gene W. Yeo

**Affiliations:** 1 Department of Physiology, Yong Loo Lin School of Medicine, National University of Singapore, Singapore, 117593 Singapore; 2 Department of Cellular and Molecular Medicine, Stem Cell Program and Institute for Genomic Medicine, University of California, San Diego, La Jolla, CA 92093 USA; 3 Molecular Engineering Laboratory, A*STAR, Singapore, 138673 Singapore

## Abstract

Mutations in genes that encode RNA-binding proteins (RBPs) have emerged as critical determinants of neurological diseases, especially motor neuron disorders such as amyotrophic lateral sclerosis (ALS). RBPs are involved in all aspects of RNA processing, controlling the life cycle of RNAs from synthesis to degradation. Hallmark features of RBPs in neuron dysfunction include misregulation of RNA processing, mislocalization of RBPs to the cytoplasm, and abnormal aggregation of RBPs. Much progress has been made in understanding how ALS-associated mutations in RBPs drive pathogenesis. Here, we focus on several key RBPs involved in ALS—TDP-43, HNRNP A2/B1, HNRNP A1, FUS, EWSR1, and TAF15—and review our current understanding of how mutations in these proteins cause disease.

## Introduction

RBPs play a central role in mediating post-transcriptional gene expression. They directly bind to RNA and control all aspects of RNA processing, including RNA synthesis, maturation, localization, translation, and decay. A genome-wide survey of RBPs in the developing mouse nervous system revealed that two-thirds of RBPs are expressed in a cell-type specific manner (McKee et al. 2005). This specialized expression of RBPs is thought to maintain a diverse gene expression profile to support the varied and complex functions of neurons. An increasing number of RBPs are being recognized as causal drivers or associated with neurological diseases (Polymenidou et al. 2012; Belzil et al. 2013; Nussbacher et al. 2015), which reinforces the importance of RBPs in maintaining normal physiology in the nervous system.

Mutations in genes that encode RBPs have been observed in patients with motor neuron disorders such as amyotrophic lateral sclerosis (ALS), spinal muscular atrophy (SMA), multisystem proteinopathy (MSP) and frontotemporal lobar degeneration (FTLD). In adults, ALS is the most common motor neuron disorder and is characterized by progressive loss of upper and lower motor neurons, which leads to fatal paralysis (Eykens and Robberecht 2015). The causes of ALS remain largely unknown, with 90% of cases being sporadic and the remaining 10% having a hereditary component (Pasinelli and Brown 2016). Model organisms (mouse, yeast, and drosophila) and human cells [patient tissue or neurons generated from patient-derived induced pluripotent stem cells (iPSCs)] have enabled significant progress in dissecting the normal and pathological functions of ALS-linked RBPs.

**Fig. 1:**
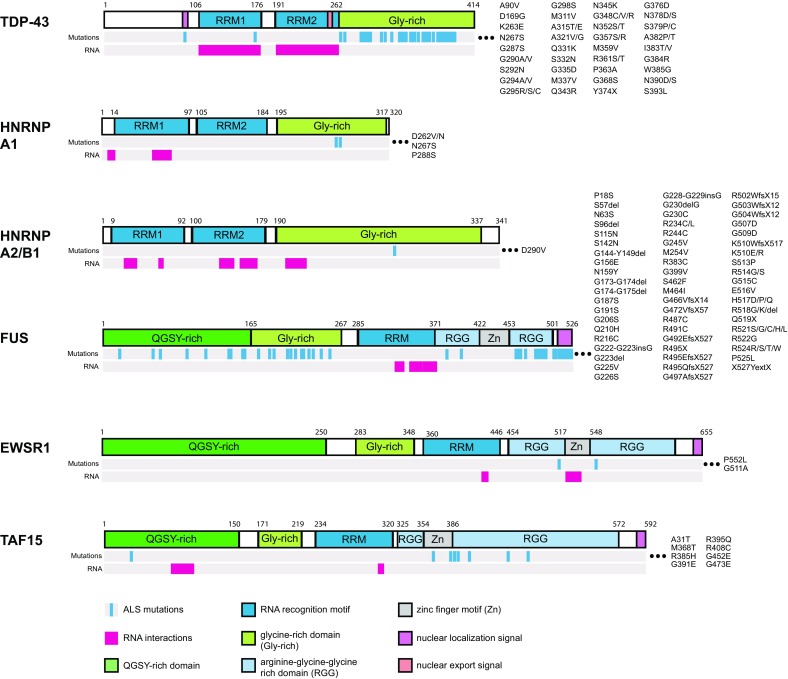
Mutation and RNA interaction maps for ALS-associated RNA-binding proteins (RBPs). The locations of mutations identified in familial and sporadic ALS patients are mapped against the domain structure of the RBP. Mutations that cause a change in the protein sequence—missense mutations, frame shifts, and deletions—are reported. Points of contact between RBPs and mRNA are shown (TDP-43, Buratti and Baralle 2001; all others, Castello et al. 2016)

**Fig. 2:**
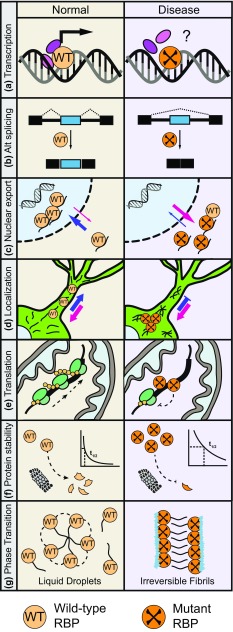
ALS-associated mutations in RBPs may disrupt RNA processing by several mechanisms. **a** Wild-type RBPs have roles in transcription. Mutant forms of the proteins may have abnormal interactions with transcription factors like TFIID or RNA polymerase II that disrupt transcription. **b** RBPs bind to introns of pre-mRNAs and splicing factors to regulate constitutive and alternative splicing. ALS-mutant proteins alter global splicing through events like exon skipping. **c** ALS-associated RBPs predominantly reside in the nucleus. Mutations in the RBPs can cause mislocalization to the cytoplasm where they bind and regulate different sets of RNAs. Some mutant RBPs were found to promote mislocalization of wild-type RBPs to the cytoplasm. **d** RBPs are involved in RNA trafficking, particularly to distant axonal and dendritic sites in neurons. Mutations in RBPs, such as FUS and TDP-43, disrupt the protein’s ability to transport mRNAs to their proper destinations. **e** TDP-43, hnRNP A2/B1, and hnRNP A1 are implicated in translation and mutant forms of these RBPs may disrupt this function. For example, mutant forms of TDP-43 have a greater propensity to mislocalize to the mitochondria and block translation of specific mitochondria-transcribed mRNAs (Wang et al. 2016a). **f** Mutations in ALS-associated RBPs, like FUS and TDP-43, cause the protein to be more resistant to proteasome-mediated degradation. The longer half-lives of mutant proteins results in their accumulation, which may confer toxicity. **g** Wild-type RBPs naturally form membrane-less organelles through phase transitions into liquid droplets. These reversible interactions are mediated by RNA and the low-complexity Gly-rich domain of the RBP. The presence of ALS-associated mutant RBPs, dipeptide repeats, or RNA repeats alter the biophysical properties of phase transitions and droplet formations. In these cases, droplets may evolve into insoluble structures through fibrilization that disrupt membrane-less organelles and kill neurons

There are more than one hundred genes associated with ALS, a handful of which encode proteins that control RNA processing (Wroe et al. 2008). Here, we focus on several RBPs that are known to be mutated in patients with ALS: TAR DNA-binding protein 43 (TDP-43), heterogeneous nuclear ribonucleoprotein A1 (hnRNP A1), heterogeneous nuclear ribonucleoprotein A2/B1 (hnRNP A2/B1), fused in sarcoma/translocated in liposarcoma (FUS/TLS, herein referred to as FUS), Ewing’s sarcoma breakpoint region 1 (EWSR1), and TAF15 (TATA-box binding protein associated factor 15) (Fig. 1). There are many commonalities between these proteins. TDP-43, hnRNP A1, and hnRNP A2/B1 are structurally similar in that they contain two RNA recognition motifs (RRMs) and a Gly-rich C-terminal domain (Fig. 1) (He and Smith 2009). FUS, EWSR1 and TAF15, which form the FET family, also share a similar structure: a zinc finger domain and RRM that facilitates DNA and RNA binding, respectively, an N-terminal low complexity, prion-like domain that mediates protein interactions and self-assembly (Han et al. 2012; Kato et al. 2012; Kwon et al. 2013; Schwartz et al. 2012), multiple C-terminal Arg-Gly-Gly (RGG) domains that facilitate non-specific RNA binding and protein interactions, and an atypical Pro-Tyr nuclear localization signal (PY-NLS) that is recognized by transportin to control nuclear–cytoplasmic shuttling (Lee et al. 2006) (Fig. 1). They are predominantly nuclear, multifunctional proteins that are widely expressed in most cell and tissue types and are implicated in a broad range of cellular processes. The importance of these proteins is exemplified by the observations that compete loss of TDP-43 or hnRNP A1 in mice is embryonic lethal (Kraemer et al. 2010; Liu et al. 2017) while complete loss of FUS or EWSR1 in mice is postnatal lethal (Hicks et al. 2000; Li et al. 2007). Disease-related mutations in these RBP have been reported to alter the normal functions of the protein in various steps of RNA processing (Fig. 2). In this review, we discuss their normal functions in RNA processing, their connection to ALS, and speculate how ALS-associated mutations in the genes that encode these proteins may contribute to pathogenesis.

## TDP-43

### Roles in RNA processing

Initially described as a transcription factor (Ou et al. 1995), TDP-43 is now better known for its role in RNA processing. Several groups have used crosslinking and immunoprecipitation followed by sequencing (CLIP-seq) to identify the global RNA targets of TDP-43 and showed that TDP-43 binds to thousands of RNAs (Tollervey et al. 2011; Xiao et al. 2011; Polymenidou et al. 2011; Sephton et al. 2011). Two RRMs in TDP-43 mediate the protein’s interaction with RNA (Fig. 1) with the first RRM (RRM1) being necessary and sufficient to bind RNA (Buratti and Baralle 2001). TDP-43 shows a strong preference for UG-repeat motifs, but also binds to non-UG sequences. A critical function of TDP-43 is controlling alternative splicing. Indeed, TDP-43 interacts with proteins involved in splicing (Freibaum et al. 2010) and a loss of TDP-43 causes widespread changes in alternative splicing (Polymenidou et al. 2011; Tollervey et al. 2011). The majority of TDP-43 binding occurs in introns (Polymenidou et al. 2011; Xiao et al. 2011; Tollervey et al. 2011), but when localized to the cytoplasm TDP-43 is mostly bound within 3′UTR of mRNAs (Colombrita et al. 2012). A concentration of TDP-43 binding sites near 5′ or 3′ splice sites of several exons was associated with both splicing repression (Buratti et al. 2001; Passoni et al. 2012; Mercado et al. 2005) and splicing activation (Fiesel et al. 2012).

Under physiological conditions, TDP-43 is predominantly nuclear but shuttles to and from the cytoplasm (Ayala et al. 2008b; Colombrita et al. 2012). Both Tollervey et al. (2011) and Colombrita et al. (2012) observed that the majority of cytoplasmic TDP-43 bound to the 3′UTRs of target mRNAs, suggesting a role in RNA transport, translation, and/or stability. Indeed, a large fraction of proteins that co-purify with TDP-43 are involved in RNA transport and translation (Freibaum et al. 2010). In neurons, the pathways that control RNA trafficking and translation are intimately linked to control the transport of select mRNAs from the soma to distal axonal and dentritic compartments, including neuromuscular junctions (Bramham and Wells 2007). While in transit, mRNAs are translationally repressed, and only upon reaching their synaptic destination are signals received that activate translation (Liu-Yesucevitz et al. 2011). TDP-43 is present in RNA granules and promotes anterograde axonal transport (from soma to axonal compartments) and translational repression for a subset of target mRNAs (Wang et al. 2008a; Alami et al. 2014). Translation is also tightly regulated during cellular stress. Stress granules are cytoplasmic protein–RNA assemblies that form in response to cellular stress and contain non-translating mRNA and various proteins, including TDP-43 (Li et al. 2013). TDP-43 associates with mRNAs that are bound by stalled ribosomes during cellular stress (Higashi et al. 2013) and this association may be mediated by the ribosomal scaffold protein RACK1 (Receptor Activated C Kinase 1) (Russo et al. 2017). TDP-43 is also reported to control RNA stability through several mechanisms. TDP-43 negatively regulates its own transcript through a mechanism that requires binding to the 3′UTR of *TDP43* mRNA to promote degradation, possibly by the exosome (Ayala et al. 2008a; Ayala et al. 2011). TDP-43 has also been shown to regulate its own expression by mediating alternative splicing of an exon in the *TDP43* mRNA 3′UTR, making the transcript a substrate for nonsense-mediated decay (Polymenidou et al. 2011).

### Disease association

TDP-43 was first associated with autosomal dominant ALS (and FTLD) in 2006. It was identified as a major component of ubiquitinated cytoplasmic inclusions in spinal motor neurons from ALS patients (Neumann et al. 2006; Arai et al. 2006). The clinical and pathological features of ALS associated with TDP-43 are highly variable, presenting as a spectrum of disorders collectively referred to as TDP-43 proteinopathies (Geser et al. 2010). Mutations in *TARDBP* were later identified in sporadic and familial ALS cases (Sreedharan et al. 2008). More than 50 missense mutations have been identified in *TARDBP* in ALS, accounting for 1–2% of total ALS cases (Van Deerlin et al. 2008; Gitcho et al. 2008; Sreedharan et al. 2008; Kabashi et al. 2008; Yokoseki et al. 2008; Millecamps et al. 2010; Corrado et al. 2009; Xiong et al. 2010; Kirby et al. 2010; Nozaki et al. 2010; Chiang et al. 2012; Orrù et al. 2012; Lemmens et al. 2009; Del Bo et al. 2009; Kamada et al. 2009; Zou et al. 2012; Iida et al. 2012; Huey et al. 2012; Rutherford et al. 2008; Kühnlein et al. 2008; Fujita et al. 2011; Ticozzi et al. 2011b; Tsai et al. 2011; Origone et al. 2010; Daoud et al. 2009; Chio et al. 2012). The majority of *TARDBP* mutations cluster in the Gly-rich domain (Fig. 1), a region that mediates protein–protein interactions (Buratti et al. 2005). The common features of TDP-43 proteinopathies are an accumulation of TDP-43 in insoluble, cytoplasmic inclusions, concomitant with a loss of nuclear TDP-43, the presence of truncated 20–25 kDa and 35 TDP-43 C-terminal fragments, and abnormal phosphorylation and ubiquitination of TDP-43 in the upper and lower motor neurons and in other regions of the central nervous system (Geser et al. 2010; Mackenzie et al. 2010).

### Mechanisms of pathogenicity

Many studies have been performed to determine how mutations in *TARDBP* cause neuron dysfunction, in particular testing the possibility of a loss of nuclear function, a gain of cytoplasmic function, or both (reviewed in Lee et al. 2012; Buratti 2015). TDP-43 has multiple roles in RNA processing that influence global changes in gene expression (Ratti and Buratti 2016), and mutations in *TARDBP* can alter gene expression, for example by affecting alternative splicing (Arnold et al. 2013; Highley et al. 2014). It is unclear whether mutant forms of TDP-43 have different affinities for target RNAs. RNA binding is required for TDP-43-mediated toxicity since mutations in TDP-43 that disrupt RNA binding (F147L and F149L) can prevent TDP-43-mediated toxicity (Voigt et al. 2010). Interestingly, ALS-associated variants of TDP-43 caused neural defects in *Drosophila* to varying degrees but did not exacerbate the neural defects caused by ectopic expression wild-type TDP-43 alone. This may reflect a dose-dependent effect of TDP-43-mediated toxicity (Voigt et al. 2010). TDP-43 variants can increase the half-life of the protein (Ling et al. 2010; Barmada et al. 2010; Watanabe et al. 2013). The exact mechanism by which mutant forms of TDP-43 affect its degradation is not clear, but there is evidence to suggest that ALS-associated mutations in *TARDBP*, most of which reside in the disordered prion-like domain, promote protein misfolding and stabilize TDP-43 within cytoplasmic inclusions (Kabashi et al. 2010; Johnson et al. 2009). TDP-43 variants affect RNA processing to varying extents and it is unclear how much of this functional variation is attributed to dosage. Several mutant forms of TDP-43 showed impaired axonal transport of RNA (Alami et al. 2014) and TDP-43 Q331K preferentially enhanced exon exclusion for select target pre-mRNAs (Arnold et al. 2013). Mutant TDP-43 protein may also influence gene expression by altering its association with other proteins. Ling et al. (2010) showed that TDP-43 Q331K and M337V mutants had enhanced binding to FUS, another RBP implicated in ALS (discussed below). TDP-43 and FUS co-regulate *HDAC6* mRNA in a manner that requires the mutation-prone Gly-rich domain of TDP-43 (Kim et al. 2010). While this is but one example of co-regulated gene expression by TDP-43 and FUS, both proteins have thousands of shared RNA targets (Lagier-Tourenne et al. 2012), and more examples of TDP-43/FUS co-regulation are likely to emerge.

Cytoplasmic mislocalization of TDP-43 is a well-established feature of neurological diseases but not an absolute requirement for cell toxicity. Some studies have shown that mutations in TDP-43 promote mislocalization to various subcellular locations in the cytoplasm (Johnson et al. 2009; Barmada et al. 2010), while other studies have shown that TDP-43 mutants are retained in the nucleus (Arnold et al. 2013). Clearance of TDP-43 from the nucleus can cause numerous downstream effects that lead to neurotoxicity. Wang et al. (2016a) showed that TDP-43 localized to mitochondria and repressed the expression of mitochondrial mRNAs. Interestingly, mutant forms of TDP-43 had increased mislocalization to the mitochondria, suggesting that mutant TDP-43 can cause greater mitochondria dysfunction (Wang et al. 2016a). In rat hippocampal neurons, cytoplasmic TDP-43 resides within RNA granules that travel to dendritic arbors upon depolarization (Wang et al. 2008a); however, ALS-associated mutant forms of TDP-43 (A315T and Q343R) severely reduced the movement of the granules to dendrites (Liu-Yesucevitz et al. 2014; Alami et al. 2014), likely preventing the transport and therefore local translation of mRNAs encoding proteins that are required for proper synaptic function. Nuclear clearance of TDP-43 into the cytoplasm is often accompanied by the formation of protein aggregates. Aggregation of TDP-43 protein is dependent on the C-terminal domain where the majority of ALS-associated mutations occur. Interestingly, Johnson et al. (2009) observed that mutant TDP-43 proteins accelerated TDP-43 aggregation in yeast; however, it is unclear if insoluble aggregates in the cytoplasm confer toxicity or are secondary effects. The accumulation of TDP-43 into insoluble cytoplasmic foci may be a consequence of nuclear clearance since abolishing one or both nuclear localization signals in TDP-43 are sufficient to cause cytoplasmic aggregation (Winton et al. 2008; Igaz et al. 2011). It is important to note that TDP-43 aggregation in the cytoplasm is not necessary to confer cellular toxicity (Barmada et al. 2010).

TDP-43 was recently reported to undergo phase transition and form liquid droplets: membrane-less organelles that contain a morphologically, physically, and functionally distinct sub-structure (Molliex et al. 2015; discussed in more detail below). Membrane-less organelles include structures such as stress granules to which TDP-43 and cellular toxicity are strongly linked (Bentmann et al. 2013; Li et al. 2013). The ability of TD-43 to form liquid droplets, at least in the nucleus, was independent of RNA binding, but rather required the prion-like QGSY-rich domain and low-complexity Gly-rich domain (Schmidt and Rohatgi 2016). Interestingly, TDP-43 variants M337V, N345K, or A382T altered the fluid characteristics of the TDP-43-containing liquid droplets. Schmidt and Rohatgi (2016) found that the exchange of TDP-43 N345K and A382T monomers between droplets and the nucleoplasm was impaired. In contrast, TDP-43 M337V monomers did not rearrange within the droplet to the same extent as wild-type TDP-43. M337V resides within a region of the low-complexity domain that forms an α-helix structure. This α-helix structure was reported to be necessary to form liquid droplets (Conicella et al. 2016) and it is possible that M337V changes the α-helix structure to alter the liquid droplet properties of TDP-43. M337V, N345V, and A382T TDP-43 variants had different effects on the phase transition properties of TDP-43 and it will be of interest to determine how other ALS-associated mutations affect the phase transition properties of TDP-43.

## HNRNP A1

### Roles in RNA processing

Heterogeneous nuclear ribonucleoprotein (hnRNP) A1 is a member of the hnRNP family, a group of related proteins known to associate with nascent mRNA (Dreyfuss et al. 1993; Jean-Philippe et al. 2013; Piñol-Roma et al. 1988). HnRNPs generally participate in many stages of the mRNA life cycle and hnRNP A1 is no exception. Previous reports have implicated hnRNP A1 in constitutive (Jurica et al. 2002; Tavanez et al. 2012; Zhou et al. 2002) and alternative splicing (Mayeda and Krainer 1992; Fisette et al. 2010). HnRNP A1 associates directly with the spliceosome as well as with a variety of exonic and intronic sequences (Han et al. 2005). Binding is typically associated with repression of nearby exons (Blanchette and Chabot 1999; Nasim et al. 2002). In certain cases, hnRNP A1 and other hnRNPs compete with serine-rich proteins to regulate alternative splicing in an opposing fashion (Kashima and Manley 2003; Cartegni and Krainer 2002; Dirksen et al. 2000; Sun et al. 1993). Synergistic control of alternative splicing has been demonstrated through direct interaction between hnRNP A1 and hnRNP H via their Gly-rich domains (Fisette et al. 2010). Similarly, functional genomic studies have revealed a broad collaboration between hnRNP A1 and other hnRNPs to control alternative splicing throughout the transcriptome (Huelga et al. 2012). Besides pre-mRNA splicing, hnRNP A1 has been linked to other mRNA processing events. HnRNP A1 is primarily nuclear, but nuclear–cytoplasmic shuttling is enabled through the M9 sequence (Mili et al. 2001; Nakielny and Dreyfuss 1999). This behavior suggests a role in nuclear export of mRNA (Gallouzi and Steitz 2001; Izaurralde et al. 1997). HnRNP A1 also plays a role in the regulation of mature mRNAs. HnRNP A1 has been reported to bind AU-rich sequences to control the stability of a number of transcripts (Henics et al. 1994; Hamilton et al. 1997). Internal Ribosomal Entry Sequence (IRES) mediated and cap-dependent translation is also regulated by hnRNP A1 (Cammas et al. 2007; Bonnal et al. 2005; Svitkin et al. 1996). Finally, hnRNP A1 regulates the processing of noncoding RNAs, especially miRNAs (Michlewski et al. 2008). HnRNP A1 was reported to specifically bind pri-miR-18a and the pri-let7a loop to either facilitate or inhibit pri-miRNA processing in a target-specific manner (Michlewski and Cáceres 2010; Guil and Cáceres 2007).

### Disease association

To date, there are three reported cases of *HNRNPA1* mutations linked to ALS or multisystem proteinopathy (MSP), a group of pleiotropic neurodegenerative disorders that includes ALS. Kim et al. (2013) described two families, one with MSP and another with ALS with the phenotype segregating according to an autosomal dominant mode of inheritance. The affected family members harbored damaging mutations in the Gly-rich domain of *HNRNPA1*. In one family affected by MSP, the *HNRNPA1* (p. D262V) mutation was identified (Fig. 1). Strikingly, this aspartic residue is homologous to the D290 residue identified in hnRNP A2/B1 that is associated with ALS (discussed below). Recently, Liu et al. (2016) sequenced a Chinese family with “Flail arm ALS” and discovered a P288S mutation (P340S in the long isoform) in *HNRNPA1* that segregated with the disease, according to an autosomal dominant mode of inheritance. In general, mutations in hnRNP A1 are not common and studies in European and other populations have failed to identify additional *HNRNPA1* variants linked to ALS. In the case of ALS, the mechanisms by which *HNRNPA1* mutations cause disease are not well understood (see below). However, in the case of Alzheimer’s disease, a simple link between hnRNP protein levels and disease has been established. Along with hnRNP A2/B1, hnRNP A1 was shown to be depleted in Alzheimer’s patient brains (Berson et al. 2012). These data linking hnRNP mutations and protein levels to multiple neurodegenerative diseases imply a strong connection between hnRNP homeostasis and neurological health.

### Mechanisms of pathogenicity

The mechanisms of pathogenicity of hnRNP A1 variants are not well studied. It is unclear if damaging mutations represent gain or loss of function or both. Mutations in the region of *HNRNPA1* that encode the Gly-rich domain are shown to strengthen “steric zippers” already present in the wild-type protein. This leads to increased fibrillization of the protein in vitro and an accumulation in stress granules in cell-based models (Kato et al. 2012; Kim et al. 2013; Molliex et al. 2015; Liu et al. 2016). HnRNP A1 has been reported to interact with other ALS-linked proteins, for example TDP-43, through its C-terminus. Honda et al. (2015) examined spinal cord motor neurons from ALS patients and found that cytoplasmic relocalization of TDP-43 was associated with loss of nuclear hnRNP A1. In another study, Gilpin et al. (2015) characterized a direct interaction between hnRNP A1 and ubiquilin-2 by yeast two-hybrid studies and other methods, showing that this interaction depended on the Gly-rich domain of hnRNP A1. Mutations in ubiquilin-2 as well as the hnRNP A1 D262V mutation reduced or eliminated hnRNP A1-ubiquilin-2 interactions. Finally, the authors demonstrated that ubiquilin-2-hnRNP A1 interactions may stabilize hnRNP A1 and increase its steady-state protein levels (Gilpin et al. 2015). Importantly, mutations in *UBQLN2* are also responsible for X-linked ALS and FTLD (Deng et al. 2011). This implies that seemingly disparate ALS proteins may in fact regulate each other through the critical Gly-rich domain. These data are compelling considering a recent study that indicated that hnRNP A1 autoregulates its own alternative splicing. HnRNP A1 protein was shown to negatively regulate *HNRNPA1* mRNA levels by inhibiting splicing of intron 10 (Suzuki and Matsuoka 2017). This mode of regulation was essential for maintaining the protein at a non-cytotoxic level. Although a definitive consensus for how mutant hnRNP A1 causes neurological dysfunction has not emerged, a significant amount of evidence has accumulated linking hnRNP A1 misfolding and fibrilization to disease. However, other studies indicating that ALS pathogenesis may simply be due to changes in hnRNP A1 levels should not be discounted. These hypotheses are not exclusive. Pathogenesis could very well result from some combination of toxic misfolded protein, reduced normal function due to accumulation in stress granules, and alterations in overall protein levels.

## HNRNP A2/B1

### Roles in RNA processing

A close relative of hnRNP A1, hnRNP A2/B1 exists in two distinct isoforms, A2 (341 amino acids) and B1 (353 amino acids), both transcribed from the *HNRNPA2B1* gene. The putative functions of hnRNP A2/B1 include pre-mRNA splicing (Clower et al. 2010; Hutchison et al. 2002), mRNA trafficking (Gao et al. 2008; Raju et al. 2011; Shan et al. 2003; Fahling et al. 2006; Goodarzi et al. 2012), and translational control (Kosturko et al. 2006). Similar to its relatives TDP-43 and hnRNP A1, hnRNP A2/B1 contains two RRMs and has a Gly-rich domain near its C-terminal end (Fig. 1). HnRNP A2/B1 is primarily localized in the nucleus; however, nuclear–cytoplasmic trafficking does occur and is controlled in part by the M9 nuclear localization signal near the Gly-rich domain (Siomi et al. 1997). A previous report has also implicated hnRNP A2/B1 in miRNA processing and biogenesis (Alarcón et al. 2015). However, subsequent studies have found only minimal effects on mature miRNA expression (Martinez et al. 2016). The role of hnRNP A2/B1 in miRNA processing remains unclear and its effects may be context or cell-type specific. MiRNAs can be sorted into exosomes and then transported to the processes of oligodendrocytes in a manner that is mediated by hnRNP A2/B1 (Villarroya-Beltri et al. 2013). Putative targets of these exosomal miRNAs included *BDNF* and *MBP* mRNAs. There is a well-characterized role for hnRNP A2/B1 in trafficking mRNAs to neuronal dendrites, where hnRNP A2/B1 recognizes a 21-nucleotide A2 response element (A2RE) in target transcripts (Raju et al. 2011; Munro et al. 1999; Shan et al. 2000, 2003). In one report, hnRNP A2/B1 binding to an A2RE in the *VEGFA* 3′UTR promoted translational read-through and consequently increased the production of *VEGF*-Ax, an anti-angiogenic form of *VEGFA* (Eswarappa et al. 2014).

### Disease association

To date, only a single mutation in *HNRNPA2B1* (p. D290V) has been reported in a family with MSP, but not specifically ALS (Kim et al. 2013). The authors described a constellation of symptoms affecting the brain, motor neurons, muscle, and bone. These symptoms were previously characterized by a disorder called inclusion body myositis, Paget’s disease of blood and bone, and frontotemporal dementia (IBMPFD), now commonly referred to as MSP. Family members were affected according to an autosomal dominant mode of inheritance. It is notable that the pathogenic D290V mutation occurred within the Gly-rich domain of hnRNP A2/B1 (Fig. 1). This is akin to TDP-43 where most ALS-causing mutations also occur in the Gly-rich domain (Lagier-Tourenne et al. 2010). *Drosophila* models expressing hnRNP A2 D290V recapitulated the myopathy and protein inclusion pathology seen in patients (Kim et al. 2013). Other groups have attempted but were unable to identify other hnRNP A2/B1 mutations in European ALS and MSP populations (Seelen et al. 2014; Le Ber et al. 2014). A large study involving exome sequencing of 2869 ALS patients also failed to find additional ALS cases with mutations in *HNRNPA2B1*. This indicates that mutations in *HNRNPA2B1* associated with ALS and MSP are exceedingly rare. Loss of hnRNP A2/B1 has been implicated in the pathogenicity of Alzheimer’s disease, although no associated genetic variants have been reported to date. Berson et al. (2012) found that depletion of hnRNP A2/B1 in mouse cortex resulted in impaired cognitive function and aberrant alternative splicing. The same study observed decreased levels of hnRNP A2/B1 and hnRNP A1 in the entorhinal cortex of patients with sporadic Alzheimer’s disease. In an in vivo experiment, ablation of cholinergic input to the entorhinal cortex resulted in reduced hnRNP A2/B1 expression and mis-splicing events consistent with those observed in post-mortem Alzheimer’s brain (Berson et al. 2012). These data link cholinergic neuron loss, a hallmark of Alzheimer’s disease, to reduced hnRNP levels and mis-splicing, which may in turn explain some of the cognitive deficits observed in Alzheimer’s disease.

### Mechanisms of pathogenicity

Investigations on the mechanisms of hnRNP A2/B1 D290V toxicity are still at an early stage. Martinez et al. (2016) showed that hnRNP A2/B1 D290V is not equivalent to loss of hnRNP A2/B1 protein, at least with regard to alternative splicing. Several studies have reported increased aggregation and localization of hnRNP A2/B1 D290V to stress granules in cell-based systems (Kim et al. 2013; Martinez et al. 2016). Martinez et al. (2016) also showed that iPSC-derived motor neurons expressing hnRNP A2/B1 D290V had increased cell death and an exacerbated stress response. These data imply that hnRNP A2/B1 D290V may be a gain-of-function mutation with toxic properties. Biochemical experiments exploring fibrillization have confirmed that hnRNP A2/B1 D290V is more prone to self-seeding compared to its wild-type counterpart (Kato et al. 2012; Kim et al. 2013; Shorter and Taylor 2013). The pathogenicity of hnRNP A2/B1 D290V may lie in the protein’s ability to form fibrils and aggregates through interactions involving its Gly-rich domain. Pathogenic aggregates of proteins typically occur in the cytoplasm; however, an increase in nuclear-insoluble hnRNP A2/B1 was observed in a subset of ALS and MSP patients (Martinez et al. 2016). This implies that aggregation and fibrillization may also occur in the nucleus and could affect alternative splicing through the assembly of aberrant ribonucleoprotein complexes. It is unclear if and how nuclear aggregation of hnRNP A2/B1 D290V accounts for the alternative splicing changes caused by the mutant protein. In the CGG expanded repeat RNA disorder Fragile X-associated tremor/ataxia syndrome (FXTAS), pathogenicity may emerge through functional sequestration of hnRNP A2/B1 by toxic RNA repeats (Muslimov et al. 2011). CGG repeats were found to directly bind hnRNP A2/B1 and lead to mis-splicing (He et al. 2014; Blanchette et al. 2009). This effect was abrogated by ectopic expression of TDP-43 and was dependent on an intact C-terminal Gly-rich domain (He et al. 2014). These data raise interesting questions regarding the relevance of RBP protein–protein interactions and RBP-repeat interactions in ALS. Indeed, the intronic GGGGCC repeat expansion in *C9orf72* is responsible for a large fraction of ALS cases (DeJesus-Hernandez et al. 2011; Renton et al. 2011). This hexanucleotide repeat is known to form G-quadruplexes that can bind hnRNP A2/B1 and hnRNP A1 (Mori et al. 2013; Sareen et al. 2013) and cause TDP-43 pathology when expressed in transgenic mice (Chew et al. 2015). Going forward, it will be important to determine how TDP-43, hnRNP A2/B1, and GGGGCC repeats interact to modulate the toxicity observed in *C9orf72* linked ALS.

In Alzheimer’s disease, loss of hnRNP A2/B1 might generate pathogenicity independently of any aggregation or fibrilization. Extending the work of Berson et al. (2012) mentioned above, Kolisnyk et al. (2016) described cholinergic regulation of hnRNP A2/B1 through the M1 muscarinic receptor. The mechanism of protein reduction was identified as differential translation efficiency due to an autoregulatory splicing event in the *HNRNPA2B1* 3′UTR. Loss of muscarinic input biased the *HNRNPA2B1* transcript toward an NMD-sensitive isoform with reduced translation efficiency (Kolisnyk et al. 2016; McGlincy et al. 2010). Importantly, the authors did not observe cytoplasmic aggregates or increased detergent insoluble fractions when they examined hnRNP A2/B1 in *Vacht*-deficient mice, indicating a pathogenic mechanism distinct from what is observed in ALS.

## FUS

### Role in RNA processing

FUS has been implicated in several RNA processing events, particularly transcription, alternative splicing, and mRNA trafficking (Lagier-Tourenne et al. 2010; Fujii and Takumi 2005). FUS carries out these functions by directly binding to RNA in a RRM-dependent manner (Daigle et al. 2013), but the Gly-rich domain may also interact with RNA (Fig. 1) (Castello et al. 2012). Using CLIP-based methods, several groups have identified the RNA targets of FUS in various contexts including human cell lines (Hoell et al. 2011), mouse cell lines (Ishigaki et al. 2012; Colombrita et al. 2012), and human or mouse brain tissue (Rogelj et al. 2012; Lagier-Tourenne et al. 2012). In two studies, FUS-binding sites were enriched for GGU motifs (Rogelj et al. 2012) or GUGGU motifs (Lagier-Tourenne et al. 2012), and a sequence preference for GGUG was been previously identified in vitro (Lerga et al. 2001). FUS appears to have limited sequence specificity, since both iCLIP and CLIP-seq studies identified a 2- to 3-fold enrichment for the GGU motif, which contrasts with a 15-fold enrichment for the GU-rich motif in TDP-43 iCLIP studies (Rogelj et al. 2012). This may explain why other studies did not identify a preferred FUS biding motif, but instead found that FUS preferred to bind RNA regions with AU-rich stem-loop structures (Hoell et al. 2011; Ishigaki et al. 2012).

The repertoire of FUS RNA targets is large with thousands of RNA targets and the RNA-binding patterns of FUS have been used to inform our understanding of its cellular functions. It was observed that FUS-binding sites frequently occurred in introns indicating a role in splicing. Indeed, a reduction in FUS caused global changes in alternative splicing (Lagier-Tourenne et al. 2012; Rogelj et al. 2012). Also, FUS interacts with several factors involved in splicing such as the U1–snRNP splicing complex, the SMN complex, SC35, SRSF1, SRSF3, and SRSF10 (Sun et al. 2015; Shang and Huang 2016). FUS-binding sites were also enriched in the 3′UTRs of target transcripts (Hoell et al. 2011; Lagier-Tourenne et al. 2012), which may allow FUS to mediate RNA localization and stability (Fujii and Takumi 2005; Kapeli et al. 2016).

FUS has additional roles in promoting transcription either by activating transcription of genes regulated by RNA polymerase II, or by blocking transcriptional repression of cyclin D1 and RNA polymerase III (Bertolotti et al. 1996; Hallier et al. 1998; Uranishi et al. 2001; Wang et al. 2015; Tan and Manley 2009; Schwartz et al. 2012; Kwon et al. 2013). FUS also has a role in maintaining genomic integrity through its interactions with PGC-1α (Sánchez-Ramos et al. 2011) and controlling the DNA damage response pathway through its interactions with CBP/p300 and HDAC1 (Wang et al. 2008b, 2013).

### Disease associations

FUS was initially identified as an oncofusion protein in human liposarcomas and later in other cancers where chromosomal rearrangements paired the *FUS* transactivation domain with transcription factors such as *CHOP* (Rabbitts et al. 1993; Crozat et al. 1993), *CREB3L2* (Panagopoulos et al. 2004), and *ERG* (Shimizu et al. 1993). Mutations in *FUS* were later reported in patients with ALS (Kwiatkowski et al. 2009; Vance et al. 2009). FUS pathology has also been observed in other neurological diseases, namely FTLD (Neumann et al. 2009a; Van Langenhove et al. 2010), essential tremor (Tio et al. 2016), and Huntington’s disease (Doi et al. 2008). Over 70 mutations in *FUS* have been identified in ALS patients, some of which are verified as disease causing (Fig. 1) (Deng et al. 2014). Interestingly, no genetic alterations in *FUS* have been reported in FTLD-FUS, a subtype of FTLD where FUS-positive inclusions are present (Neumann et al. 2009b; Rohrer et al. 2011; Urwin et al. 2010; Snowden et al. 2011). The pathological phenotypes caused by mutant forms of FUS vary widely in ALS patients with different mutations and even in ALS patients with the same mutation. A feature of FUS-related neuropathies, including ALS, is mislocalization of FUS to the cytoplasm either within intracellular protein aggregates or diffused throughout the region. FUS can be present in various types of protein aggregates including stress granules, ubiquitin-positive inclusions, and p62-positive inclusions (Kwiatkowski et al. 2009; Vance et al. 2009). The presence of FUS aggregates stratifies patients into a FUS-positive ALS (ALS-FUS) subtype (Woulfe et al. 2010; Deng et al. 2010).

### Mechanisms of pathogenicity

It is unclear how ALS-associated mutations in *FUS* lead to neuron dysfunction. Numerous loss- and gain-of-function studies have been performed in mice, zebrafish, rats, worms, and fly to understand how disruption of FUS activity affects normal cell physiology (reviewed in Shang and Huang 2016). Data from transgenic mouse studies generally agree that expressing mutant FUS proteins is sufficient to cause motor neuron degeneration (Nolan et al. 2016). While loss of FUS in mice causes perinatal lethality, conditional loss of FUS in the nervous system does not cause motor system dysfunction (Sharma et al. 2016). This suggests that mutant FUS protein is not equivalent to a loss of FUS function. It is likely that mutant FUS proteins instead have acquired abnormal functions perhaps by some combination of disrupting RNA and protein homeostasis, altering subcellular localization, or promoting toxic aggregates.

The pathological activity of mutant FUS depends on its ability to bind RNA. ALS-associated FUS mutants in which the RRM domain was deleted could no longer cause neurological defects in *Drosophila* (Daigle et al. 2013). Although ALS-associated mutations in *FUS* do not reside within the regions that make direct contact with RNA, the mutations could affect the ability of FUS to interact with RNA. FUS R521C formed more stable complexes with *Bdnf* mRNA compared to wild-type FUS (Qiu et al. 2014). Since mutant forms of FUS are prone to mislocalize to the cytoplasm (Dormann et al. 2010), they are exposed to different sets of RNA substrates, particularly pre-mRNA versus mature RNA. Indeed, Hoell et al. (2011) found that the majority of binding sites for wild-type FUS resided in introns, whereas binding sites for ALS-mutant forms of FUS were located in 3′UTRs. Furthermore, FUS downregulates its own expression by binding to exon 7 of *FUS* mRNA and causing exon skipping (Zhou et al. 2013). The alternatively spliced product is a *FUS* transcript that undergoes nonsense-mediated decay. FUS variants, on the other hand, were unable to induce exon 7 skipping in *FUS* and therefore caused an upregulation in FUS protein; this misregulation led to an accumulation of the protein and may be a pathogenic mechanism.

FUS interacts with numerous proteins, many of which are involved in RNA metabolism (Sun et al. 2015; Kamelgarn et al. 2016). Kamelgarn et al. (2016) performed a proteomics study to determine whether FUS R521G exhibited different protein interactions from wild-type FUS and found little difference. These studies were performed in HEK293 cells at steady state. In contrast, mutant FUS proteins were reported to have stronger interactions with Survival of Motor Neuron (SMN) than wild-type FUS (Groen et al. 2013; Sun et al. 2015). The interaction between FUS and SMN is of note since mutations in *SMN1* cause a reduction in SMN protein and leads to SMA, a degenerative disease of the lower motor neurons (Chari et al. 2009). This enhanced mutant FUS–SMN interaction was reported to alter global changes in alternative splicing by reducing the number of SMN-positive intranuclear bodies, called Gems, where spliceosome complexes are assembled (Sun et al. 2015) and impair the transport of SMN to axons (Groen et al. 2013).

The majority of *FUS* mutations associated with ALS cluster in the NLS and the N-terminal QGSY-rich and RGG1 prion-like domains (Fig. 1). These prion-like domains make FUS inherently prone to aggregation (Cushman et al. 2010). Some of these *FUS* variants, like R521C and P525L, appear to further exacerbate FUS aggregation, which is consistent with the notion that redistribution of FUS to the cytoplasm is a pathological event (Dormann et al. 2010; Bosco et al. 2010; Mackenzie et al. 2011; Gal et al. 2011; Ito et al. 2011; Kino et al. 2011; Niu et al. 2012; Suzuki et al. 2012; Zhang and Chook 2012). Mutations in *FUS* have so far only been observed in ALS, but not FTLD patients with FUS pathology (ALS-*FUS* and FTLD-FUS, respectively) (Neumann et al. 2009b; Rohrer et al. 2011; Urwin et al. 2010; Snowden et al. 2011). Neumann et al. (2011) showed that FUS-positive inclusions in ALS-*FUS* do not contain the other FET family members EWSR1 and TAF15. In contrast, FUS-positive inclusions in FTLD-FUS also contain EWSR1 and TAF15. These differences in pathological features indicate that there is a general breakdown of the nuclear import pathway in FTLD-FUS, whereas in ALS-*FUS* there is a defect specifically in FUS. Mutations in the NLS of *FUS* may impair nuclear import by weakening the interaction between FUS and transportin (Trn1) (Dormann et al. 2010; Niu et al. 2012). Trn1 interacts with the NLS of FUS to mediate its import into the nucleus. Although previous studies reported that nuclear FUS does not incorporate into stress granules, mutant FUS can bind and sequester nuclear localized FUS to cytoplasmic stress granules (Vance et al. 2013).

## EWSR1

### Role in RNA processing

EWSR1 is essential for diverse cellular processes including meiosis and mitosis (Li et al. 2007; Azuma et al. 2007), hematopoiesis and B lymphocyte development (Cho et al. 2011; Li et al. 2007), and adipogenesis (Park et al. 2013; Park and Lee 2015). Loss of EWSR1 in mice causes cellular senescence of hematopoietic stem cells (Cho et al. 2011) and genomic instability in cell types that undergo physiological DNA breaks, such as B cells and meiotic germ cells (Li et al. 2007).

Compared to FUS, less is known about the role of EWSR1 in RNA processing. The zinc finger and RRM domains of EWSR1 make contact with RNA (Fig. 1) (Castello et al. 2016). To begin understanding the role of EWSR1 in RNA processing, two groups independently mapped the RNA-binding sites of EWSR1 using PAR-CLIP in human cell lines. EWSR1 bound broadly across pre-mRNA transcripts, especially within introns, rather than at distinct sites (Mittal et al. 2015; Hoell et al. 2011). This binding profile is reminiscent of FUS, which also binds RNA broadly and is enriched in introns (Lagier-Tourenne et al. 2012; Rogelj et al. 2012). More specifically, Mittal et al. (2015) observed that EWSR1 preferred to bind G-rich RNA motifs in mRNA, consistent with previous in vitro binding studies. In contrast, Hoell et al. (2011) found that EWSR1 tended to interact with stem-loop structures. These discrepancies may be attributed to differences in how the CLIP libraries were prepared and could introduce biases in EWSR1 RNA-binding site identification.

The majority of proteins that interact with EWSR1 appear to be other RBPs that are involved in transcription, splicing, and translation (Pahlich et al. 2009). Some of these EWSR1 interactions are direct and robust, for example with FUS, TAF15, and EWSR1 itself (Thomsen et al. 2013), while other interactions are dependent on RNA (i.e., indirect interaction), like hnRNP M, hnRNP U, RNA helicases p68 and p72, the hsRPB7 subunit of RNAPII, and splicing factor YB-1 (Petermann et al. 1998; Chansky et al. 2001; Pahlich et al. 2009). EWSR1 is required for homologous recombination during the DNA damage response (Li et al. 2007) and regulates mitosis, in part through interactions with α-tubulin (Wang et al. 2016b). Further investigation is needed to determine how EWSR1 is involved in these processes and whether EWSR1 somehow connects the DNA damage response with RNA processing.

### Disease association

A role for EWSR1 in human disease is well established in the context of cancer, particularly in Ewing’s sarcoma from which the gene derives its name. The common oncogenic event involves a chromosomal rearrangement that positions the DNA transactivation/low-complexity domain of *EWSR1* upstream of DNA-binding domains of numerous genes, including *FLI*-*1*, *ATF*-*1*, and *ERG*, to form fusion oncogenes (Fisher 2014). The emergence of pathogenic mutations in *FUS* and *TDP43* and the propensity of FUS and TDP-43 proteins to form pathological aggregates in patients with ALS suggest that defects in RNA regulation may contribute to ALS pathogenesis. This motivated a search for proteins with similar features to FUS and TDP-43, namely the presence of RRMs and prion-like domains, to identify novel ALS candidate genes (Couthouis et al. 2011). EWSR1 emerged as a top candidate. Ticozzi et al. (2011a) screened the entire coding region of *EWSR1* in ALS patient and healthy control samples, but did not find any mutations that altered the protein sequence of EWSR1. Couthouis et al. (2012) also scanned the *EWSR1* gene for mutations in a different cohort of ALS patients, this time focusing only on the exons that encoded the C-terminal RGG- and PY-motif regions, as the equivalent regions in *FUS* and *TDP43* are hotspots for ALS-associated mutations. They identified two missense mutations—G511A and P552L—that were present in two unrelated sporadic ALS patients and not in control samples (Couthouis et al. 2012).

### Mechanisms of pathogenicity

G511A and P552L variants in *EWSR1* reside in the RGG domains (Fig. 1). Interestingly, these mutant EWSR1 proteins accumulated in the cytoplasm of ESC-derived neurons and in the neurites of cultured mouse spinal cord neurons, while the wild-type form of EWSR1 remained in the nucleus. The relocation of pathological forms of RBPs from the nucleus to the cytoplasm is a common feature in ALS patients. Indeed, EWSR1 protein was localized diffusely or within punctate granular structures in the cytoplasm of motor neurons from sporadic ALS patient samples but not healthy control samples. Furthermore, Couthouis et al. (2012) evaluated whether EWSR1 mutants were prone to aggregation and if they caused a neurodegeneration phenotype in the drosophila nervous system. The prion-like domain in EWSR1 (amino acids 1–280) makes the protein intrinsically prone to aggregation, but EWSR1 G511A and P552L mutants formed aggregates in vitro more rapidly than wild-type EWSR1. Wild-type EWSR1 alone was sufficient to cause neurodegeneration phenotypes when overexpressed in the fly retina and shortened the lifespan of *Drosophila* when expressed throughout the nervous system. One way in which mutations in *EWSR1* may confer toxicity is by increasing the total abundance of EWSR1 since overexpression of the wild-type protein alone is toxic. Overexpression of EWSR1 G511A or P552L mutants did not exacerbate these phenotypes. These findings imply that the wild-type version of EWSR1 has intrinsic pathogenic properties, but this does not discount the possibility that the two ALS-associated mutant forms of EWSR1 may confer additional pathogenic properties in humans.

## Taf15

### Role in RNA processing

Like its family members FUS and EWSR1, TAF15 is involved in several steps of RNA processing. TAF15 was initially discovered as a component of the TFIID complex (Bertolotti et al. 1996) and later Kwon et al. (2013) showed that TAF15 interacts specifically with the unphosphorylated form of RNA polymerase II via its low-complexity domain. The unphosphorylated form of RNA polymerase II is associated with the pre-initiation complex. A prevailing hypothesis is that TAF15, likely in complex with TFIID, recruits RNA polymerase II to sites of active transcription. TAF15 also interacts with components of the spliceosome and auxiliary proteins involved in splicing. TAF15 was reported to co-immunoprecipitate with components of the U1–snRNP complex and splicing-associated hnRNP M. These interactions required the N-terminal, low-complexity domain, but not the C-terminal domain, of TAF15 (Leichter et al. 2011; Marko et al. 2014). CLIP-seq binding studies have revealed that TAF15 binds to long introns of a subset of pre-mRNAs in a “saw tooth-like” pattern (Kapeli et al. 2016) similar to FUS (Lagier-Tourenne et al. 2012). This binding profile suggests that TAF15 is deposited onto nascent transcripts as they emerge from the RNA polymerase II complex. TAF15 also binds to the 3′UTR of select mRNAs. In fact, TAF15-binding sites were most highly enriched in the 3′UTRs of its target RNAs when the length of the different genic regions was accounted for. This implies a role in RNA stability (Kapeli et al. 2016).

Interestingly, TAF15 and FUS bind to a large number of similar RNA targets (Ibrahim et al. 2013; Kapeli et al. 2016), indicating that they regulate common networks. For a large number of these common RNA targets, TAF15 and FUS-binding sites are in close proximity (Kapeli et al. 2016). Since TAF15 and FUS proteins strongly interact with each other in a manner that is not dependent on RNA (Thomsen et al. 2013; Sun et al. 2015), it is conceivable that TAF15 and FUS can bind RNA simultaneously.

### Disease associations

TAF15 was initially associated with cancer, specifically extraskeletal myxoid chondrosarcoma and acute leukemia, in which the N-terminal transactivation domain of *TAF15* was translocated and fused to the DNA-binding domain of *NR4A3* or *CIZ*/*MNP4*, respectively (Sjögren et al. 1999; Martini et al. 2002). After connections between TDP-43 and FUS with ALS were established, TAF15 became a prime ALS gene candidate (Ticozzi et al. 2011a; Couthouis et al. 2011). Ticozzi and colleagues scanned the entire coding region of *TAF15* in patients with sporadic and familial ALS and discovered two missense mutations—A31T and R395Q—that were present in familial ALS but not healthy control patient samples (Ticozzi et al. 2011a). At the same time, Couthouis et al. (2011) had searched specifically within the C-terminal zinc finger and RGG domains of *TAF15* (not the entire gene) in 735 individuals diagnosed with ALS and discovered several mutations in patients with familial ALS but not in healthy individuals: M368T, G391E, R408C, and G473E (Fig. 1).

### Mechanisms of pathogenicity

Wild-type TAF15 is pathogenic when present at high levels. Overexpression of wild-type TAF15 is toxic in a *Drosophila* model of neurodegeneration (Couthouis et al. 2011). Furthermore, wild-type TAF15 was mislocalized to the cytoplasm in motor neurons isolated from tissues of deceased sporadic ALS individuals. TAF15 is inherently prone to aggregation as a result of having low-complexity, prion-like domains, and several studies point to this feature as mediating the protein’s pathogenic activity (Couthouis et al. 2011; Kwon et al. 2013). However, ALS-mutant forms of TAF15 can enhance the pathogenic phenotypes of TAF15 over that of wild-type protein. The presence of ALS-linked mutations in *TAF15* (G391E, R408C, and G473E) increased the number of TAF15-positive cytoplasmic foci in the dendrites and axons of embryonic rat cultured neurons above wild-type levels (Couthouis et al. 2011). Furthermore, overexpression of TAF15 G391E and R408C in *Drosophila* decreased lifespan compared to wild-type TAF15. How these mutations promote a degenerative phenotype is still unknown.

Mutant forms of TAF15 may alter the normal activities of TAF15 by disrupting RNA or protein interactions. Mutations in TAF15 do not reside in the RRM of TAF15, the domain that facilitates RNA binding, and so may not perturb TAF15–RNA interactions. Instead, ALS-mutant forms of TAF15 may alter the subcellular localization of TAF15, and as a result prevent nuclear functions of TAF15, promote toxic cytoplasmic functions, or both. Indeed, ALS-mutant forms of TAF15, namely G391E, R408C, and G473E, had a higher propensity to localize to cytoplasmic foci (Couthouis et al. 2011). One mechanism that controls TAF15 subcellular localization involves methylation by PRMT1 (protein arginine methyltransferase 1). When TAF15 methylation was blocked by a chemical inhibitor, a fraction of TAF15 accumulated into TIA1-positive stress granules in the cytoplasm (Jobert et al. 2009). This suggests that methylation of TAF15, presumably by PRMT1, retains the protein in the nucleus. TAF15 contains many arginine residues that are potential substrates for methylation, but R408 does not appear to be a substrate; however, the arginine residue adjacent to G473 is methylated. Further studies are needed to determine whether mutations in TAF15 alter PRMT1-mediated localization of TAF15.

## Structural and functional commonalities: implications for unifying mechanisms of pathogenicity

### Converging properties in function through protein and RNA interactions

The structural and functional commonalities between ALS-associated RBPs might suggest that they cause disease in a similar manner. As we perform more protein–protein interaction studies, we find that ALS-associated RBPs are often physically connected indicating that they operate in the same pathways. For example, hnRNP A1 and hnRNP A2/B1 interact with TDP-43 to control many of the splicing inhibitory functions of TDP-43 (Buratti et al. 2005; D’Ambrogio et al. 2009). In a proteomics study, FUS and TAF15 were identified as TDP-43 binding partners (Ling et al. 2010) and the FUS–TDP-43 interaction is required to mediate HDAC6 expression (Kim et al. 2010). Several studies have described strong interactions between FUS, EWSR1, and TAF15 (Thomsen et al. 2013; Sun et al. 2015; Kapeli et al. 2016), but the consequences of these interactions on RNA processing is unknown. FET proteins associate with a common set of proteins. For example, all FET proteins associated with TFIID and RNA Pol II to promote transcriptional activation (Bertolotti et al. 1996; Kwon et al. 2013). FUS and EWSR1 interact with a common set of splicing factors such as YB-1, U1C, SR, and SF1 (Chansky et al. 2001; Kovar 2011).

ALS-associated RBPs can also indirectly interact with each other by binding to the same RNA molecule. As RBPs typically have hundreds to thousands of RNA targets, they are likely to have overlapping RNA targets. CLIP studies that are performed under the same conditions (e.g., model system and CLIP protocol) can offer insight into common RNA targets. PAR-CLIP studies performed for FET proteins in human cell lines showed that the family members bind many of the same mRNAs: the RNA targets of FUS, EWSR1, and TAF15 overlapped with ~32, 69, and 48%, respectively, of RNA targets of the other two members (Hoell et al. 2011). In a separate CLIP-seq study performed in the mouse brain, FUS, TAF15, and TDP-43 shared a large percentage (>80%) of RNA targets (Kapeli et al. 2016). Interestingly, in one-third of TAF15 RNA targets there was at least one occurrence of a TAF15 and FUS-binding site within 100 bp of each other (Kapeli et al. 2016). Whether TAF15 and FUS bind the same RNA target in a mutually inclusive or exclusive fashion remains unknown. Genome-wide CLIP studies that define the spatiotemporal binding properties for ALS-associated RBPs with respect to each other will help to reveal new mechanisms of co-regulation between these RBPs (Fig. 2).

### Converging properties in low-complexity domains and liquid droplets

Recent trends in biology have highlighted the importance of membrane-less organelles in diverse cellular processes (reviewed in Banani et al. 2017). These molecular assemblies form dynamic micron-scale environments that mediate many biochemical reactions including those critical for RNA metabolism. The specialized RNP granules are dense collections of RNA and RBPs that exist in a variety of different subtypes including nucleoli, processing bodies (P-bodies), Cajal bodies, germ bodies, and stress granules. Much effort has focused on determining how these granules are formed and stabilized. Recent data indicate a mechanism based on the phase separation of liquid droplets containing high concentrations of RNA and RBPs (Brangwynne et al. 2009, 2011; Kato et al. 2012; Hyman et al. 2014). Indeed many, membrane-less organelles seem to be liquid-like in their behavior: dispersing and reforming, fragmenting, merging, and deforming in response to mechanical stress (Elbaum-Garfinkle et al. 2015; Weber and Brangwynne 2015). Many of the RBPs discussed in this review have been shown to undergo phase transitions and form liquid-like droplets in vitro and in vivo (Molliex et al. 2015; Patel et al. 2015; Kato et al. 2012; Schmidt and Rohatgi 2016; Paul et al. 2017). Importantly, for some RBPs, the presence of ALS-associated mutations seems to alter their phase transitions, stabilize and accelerate the formation of irreversible gel-like states, and in some cases, alter the dynamics of stress granules in cells (Lin et al. 2015; Molliex et al. 2015; Patel et al. 2015). For instance, the ALS-associated D290V mutation in hnRNP A2/B1 was shown to increase the formation of amyloid-like fibrils in vitro (Paul et al. 2017). ALS-associated mutations in hnRNP A1 were similarly shown to increase fibrilization (Molliex et al. 2015). FUS was also shown to form liquid-like droplets in vitro and in vivo and disease-causing mutations led to the formation of an irreversible solid state, not unlike the dense granules observed in post-mortem tissue obtained from ALS patients (Patel et al. 2015). In another study, mutant FUS was shown to accumulate in and disrupt cytoplasmic RNP granules and reduce new protein translation (Murakami et al. 2015). In nearly all cases, the authors demonstrated the requirement of the Gly-rich portion of RBPs for generating liquid droplets and fibrils (Lin et al. 2015; Molliex et al. 2015; Patel et al. 2015). Similarly, individual RBPs may recruit different proteins with Gly-rich domains to liquid droplets in a concentration-dependent manner (Lin et al. 2015; Molliex et al. 2015). Interestingly, the presence of RNA greatly diminishes the threshold necessary for phase transition and droplet formation (Lin et al. 2015).

A handful of recent studies have now established the importance of phase transitions and liquid droplet formation in ALS cases where mutant forms of RBPs were not involved. Dipeptide repeats generated from expanded GGGGCC repeats in C9orf72 ALS were shown to undergo phase transitions and form liquid-like droplets in vitro (Boeynaems et al. 2017; Lee et al. 2016). Dipeptide repeats were also shown to accumulate in stress granules, Cajal bodies, nuclear speckles, and the nucleolus of cells. These peptides reduced protein translation and disrupted stress granule dynamics (Boeynaems et al. 2017; Lee et al. 2016). In both studies, the authors also showed that dipeptide repeats can interact with the Gly-rich domain of several RBPs (hnRNP A1, FUS, TIA-1) and alter their phase transitions, making them more likely to remain in liquid droplets, or form fibrils (Boeynaems et al. 2017; Lee et al. 2016). Very recently, one study demonstrated that GGGGCC repeat containing RNA as well as other RNA repeats can undergo phase transitions and form liquid droplets even when only purified RNA is present (i.e., no RBPs) (Jain and Vale 2017). How the presence of Gly-rich domain containing RBPs might affect this process has not yet been reported.

In summary, the discovery of phase transitions in ALS-associated RBPs is an important step forward in understanding the pathogenic mechanisms of ALS. The observation that GGGGCC-containing RNA can form similar liquid-like droplets further strengthens the hypothesis of a common disease mechanism underlying the varied genetic causes of ALS. The disruption of endogenous membrane-less organelles by ALS proteins or RNA repeats presents a compelling biochemical mechanism for toxicity and a potential strategy for treatment.

## Conclusion

It is clear that misregulation of RNA processing caused by defects in RBPs in the nervous system contributes to neurodegeneration. The field continues to accumulate evidence that uncovers how ALS-associated mutant forms of RBPs lead to a loss or gain of function and, importantly, to discern which of these functions are causes or consequences of pathogenesis. The normal and disease forms of these RBPs are often studied in isolation. For the disease-associated RBPs that we have discussed and several that we did not discuss (e.g., TIA-1 and MATRIN 3), there are clear overlaps in physiological and pathological functions. Considering how the functions of these proteins relate to the more global properties of the nerves highlights one of the more perplexing conundrums in the field of ALS: why the neuron? Indeed, all of the proteins discussed here are ubiquitously expressed, and yet patients typically exhibit symptoms in only a few specific tissues, usually the central nervous system. By considering the properties of ALS-linked RBPs, we can speculate on why this may be the case.

We propose two non-exclusive hypotheses. First, nearly every protein discussed above is involved in RNA transport. The upper and lower motor neurons are the longest cells in the body and proper function requires the transport of RNP granules over nearly a meter of distance. Perhaps, due to their unique physiology, motor neurons are most susceptible to subtle disturbances in the RNA transport machinery. Second, most of the proteins discussed in this review are directly or indirectly involved in RNA splicing. RNA splicing is most prevalent in cells of the nervous system than in any other cell types (Yeo et al. 2004; Castle et al. 2008), resulting in a greater diversity of gene expression through alternatively spliced isoforms. If proper function of the central nervous system requires fine-tuning of alternative splicing, then cells of the brain and spinal cord may be extremely sensitive to alterations in RNA splicing, or other RNA processing events, that go unnoticed in other tissues.
